# Lessons Learned: Forty Years of Clinical Work With Suicide Loss Survivors

**DOI:** 10.3389/fpsyg.2020.00766

**Published:** 2020-04-29

**Authors:** John R. Jordan

**Affiliations:** Private Practitioner, Pawtucket, RI, United States

**Keywords:** intervention, suicide loss survivors, grief therapy, grief counseling, suicide bereavement, grief after suicide

## Abstract

The author has been a grief therapist in private practice for almost 40 years. The largest percentage of his clients have been suicide loss survivors, and in this article, the author reflects on the “lessons learned” about how grief therapy with survivors is both the same as, and very different from, work with clients bereaved after other types of losses. After briefly reviewing some of the empirical literature about differences between suicide bereavement and grief after other modes of death, the author argues that perhaps the most distinguishing and difficult aspect of a suicide loss is the “perceived intentionality” of the death, and the related “perceived responsibility” for the death. The author goes on to identify a number of tasks of psychological reintegration after a suicide loss that can serve as a template for treatment goals for clinicians and clients alike. These include the cultivation of a very specific type of secure and nurturing therapeutic alliance; extensive psychoeducation about suicide, trauma, and grief; the need to help the client repair the psychological continuing bond with the deceased; and providing gentle support for the survivor in rebuilding an assumptive world that has been shattered by the suicide of a loved one. Finally, the article concludes with a discussion of the clinical implications of these differences for work with suicide loss survivors.

## Introduction

As a clinical psychologist and grief therapist in private practice for most of my career, I have had the opportunity to know and work with many people who have been bereaved by suicide. A percentage of them had already seen another mental health clinician, before they got to me. Consequently, I have heard many stories about positive and healing experiences that survivors have had with other therapists. Unfortunately, I have also heard far too many “horror stories” about therapists who (mostly out of ignorance about what is normative after a suicide), have been at best unhelpful, and at worst, overtly injurious to the survivors. As one example, I worked with a woman who lost her adult daughter to suicide. She went to see a clinician who was recommended by her primary care physician, and after three sessions with the client, the therapist stated that “You’re dwelling on this too much,” and began trying to administer the cognitive behavioral therapy in which she had been trained. Her goal was to help her “……change your negative thinking about this loss.” *In my opinion, this example illustrates a lack of experience, knowledge, and, quite likely, training about working with newly bereaved suicide loss survivors. A very common clinical error is to immediately try to “fix” the presenting problem with the tools available to the clinician, without establishing a secure, attachment based relationship with the client, and without understanding the wide-ranging and long term impact that the loss of a child to suicide can have for mothers. This type of profound loss is, in my experience, transformational for suicide loss survivors, and is virtually always a long and slow adaptational process that does not lend itself to “quick fixes.”* I am writing this article with the hope of sharing with other professionals some of what I feel that I have learned over nearly 40 years of providing grief therapy to suicide loss survivors. My goal is not to critique other hard-working and well-intended caregiver peers. Nor is it to proselytize for the one true and correct way to work with survivors (indeed, the empirical evidence base for working with all traumatic losses such as suicide is, in my opinion, woefully under-researched (Jordan and McMenamy, 2004; McDaid et al., 2008; Andriessen et al., 2019). Rather, my hope is that this article will serve as a catalyst for my colleagues to approach the complex phenomena of suicide bereavement with the sense of respect and humility that the subject deserves, and to better educate themselves about what seems to be helpful in most clinical situations.

## Is Suicide Bereavement Different?

The question of whether, and in what ways, grief after suicide might be different from grief after other modes of death is not a new one. Several research and theoretical articles have attempted to answer this question (Jordan, 2001; Sveen and Walby, 2008; Jordan and McIntosh, 2011). Typically, the research and clinical literature has generally (although not always) found the mourning process after a suicide to differ from more normative causes of death in the following ways:

• There is a greater need to seek an explanation for the death and to make sense of the death.
• Survivors experience greater levels of guilt and felt responsibility for the death (or at a minimum, for a failure to somehow foresee and prevent the suicide).
• There is a greater level of stigmatization and shame about this mode of death, and a greater need to conceal the fact that the death was a suicide.
• Survivors receive more avoidance by, and isolation from, social support from their regular social networks.
• Exposure to the loss of a loved one to suicide increases the chances of suicidal thinking and behavior in the person exposed.

In addition, like other sudden, unexpected, and often violent deaths (such as homicides, motor vehicle crashes, and natural disasters), suicide also seems to produce higher levels of PTSD type symptoms (intrusive reliving, avoidance of triggering reminders of the death, and physiological hyperarousal; Kaltman and Bonanno, 2003; Bonanno et al., 2007; Neria et al., 2007). This is often accompanied by a significant disruption of the survivors’ assumptive world (e.g., beliefs such as “my life is predictable” and “I can keep my loved ones safe from harm”; Wickie and Marwit, 2000; Parkes, 2001; Parkes, 2013).

## What Makes Suicide Bereavement Different? the “Why?” and “Responsibility?” Questions

There are some universals in grief, no matter what the mode of death was. For example, when we lose someone important to us through death, we yearn to have them back. Yearning can perhaps be considered the hallmark of the grief response, and its presence is usually expected by both the mourner, and those in the social network around the bereaved individual. Indeed, its unremitting persistence over time is one of the signal indicators of complicated grief, since over time, most yearning for the deceased begins to gradually subside (Pearlman et al., 2014; Mauro et al., 2018).

Beyond the aspects of grieving that apply to all bereavement situations, there are several aspects of a suicide death that can be considered to be either unique to suicide, or at least much more prominent after a suicide, and other traumatic deaths. By traumatic deaths, I mean a mode of death that is sudden, unexpected, and often times violent – and thus likely to leave the mourner in a state of shock and bewilderment.

Perhaps the element that most distinguishes a death by suicide from all other deaths is the *perceived intentionality* of the death (Survivors of Suicide Loss Task Force, 2015; Jordan and McGann, 2017). That is, most survivors seem to view suicide as a voluntary choice made by the deceased to die. This perception of self-volition *by the deceased* in the cause of death is unique to suicide (The question of whether suicide is “actually” a choice, freely made by the decedent, is a complex and debatable one, and is beyond the scope of this article. What matters here is the degree to which the mourner *perceives* the death to have been a choice, versus a behavioral act to which the individual was “driven” by circumstances beyond their control). Whenever a mourner believes that someone intended a death to happen, this belief seems likely to add an extra layer of guilt and rage to the emotional response to the death. To the degree that a suicide death is also perceived as intended, then, it raises profound meaning-making and existential questions for the mourner. Why would they choose to do this? How could they have overcome their fear of death, their responsibilities, and their love for others to engage in this behavior? If this death was chosen, then could the deceased, or myself, or someone else have prevented that choice? Why wasn’t my relationship with the person enough reason to stay alive? Whose “fault” was the death, and who should be held accountable for it? To a greater or lesser degree, these are the questions with which most suicide loss survivors wrestle. They are also questions that often do not have simple or socially consensual answers, which can create a high level of angst on the part of many survivors, and misunderstanding on the part of the social network.

Closely related to the “Why?” question is the “Responsibility?” question: Who is to be held accountable for this death? This is true because, for most of the public, suicide is a mysterious and even baffling cause of death. Thus, most survivors do not have the kind of generic explanations for the death that are already socially constructed for most other modes of death. For example, the mourner can explain a death to lung cancer by the fact that the deceased was a smoker, and couldn’t or wouldn’t stop. Even a traumatic and violent death of a loved one, such as a homicide, might be explained by the evil or revengeful intent of the murderer. But why does someone choose to “murder” themselves (The Latin root of the word suicide literally means “self-killing or self-murder”)? Most people have no easy, socially validated explanation for suicidal behavior, since it seems to violate a belief that most people take for granted, which is that *“Of course, everyone wants to keep living, don’t they?”* And because we collectively do not have a commonly accepted narrative for self-inflicted death, many survivors struggle with who or what should be held accountable for the death?

While it might seem obvious that the individual who killed themselves should be held responsible, in my clinical experience, this only happens some of the time, and for only some of the survivors of a suicide loss. Instead, most survivors begin by blaming themselves for the death. Many survivors repeatedly review a litany of their own “sins of omission and commission” in trying to assign responsibility for the death. Sometimes referred to as the “if-onlys,” survivors may have thoughts such as “if only I had not had an argument with him the night before,” or “if only I had made him go to therapy or Alcoholics Anonymous, or church,” etc. Sometimes, survivors will also assign the blame to someone else, such as other family members, friends, or professionals who are deemed to have been professionally responsible for the deceased (e.g., a therapists, doctors, or clergy persons). In my experience, it is less common for survivors to begin by blaming the deceased, although that may come in time.

## Tasks of Psychological Integration of the Loss

Sometimes referred to as grief work, I have come to believe that there are several psychological tasks that most survivors need to address if they are to integrate and make their peace with the suicide. These tasks can serve as a broad guide for both the clinician and the client as to the work that needs to be done in the treatment. I will list and comment on these tasks in the bulleted list below.

• Containment of the Trauma and Restoration of a Sense of Psychological Safety and Control

Evidence suggests that exposure to the violent death of a loved one, particularly if the survivor was an eye-witness to the dying process, or found the body of the deceased, can generate trauma symptoms in the survivor (Kristensen et al., 2012; De Leo et al., 2014; Rando, 2015). *In my opinion, the core of the trauma response is elicited by exposure to a terrifying or horrifying situation, which results in intense physiological arousal, as well as a profound sense of helplessness and a loss of one’s subjective sense of predictability and control over the world. This, in turn, makes the world a significantly less psychologically safe place to dwell.* Trauma symptoms can include intrusive reliving of the sensory data from the scene (sights, sounds, smells, etc.), and strenuous attempts to avoid anything that will trigger *an involuntary relieving of the original* traumatogenic experience (*a.k.a. flashbacks*), along with efforts to suppress the physiological arousal that goes with these memories. As an example, I worked with a couple whose adolescent daughter killed herself after an intense argument with her parents. Her mother found her daughter’s body in the girl’s bedroom upstairs, and for several weeks after this trauma, she had difficulty leaving her own bedroom, and absolutely refused to go upstairs or to go into her daughter’s bedroom, *in an obvious attempt to avoid being triggered into reliving the nightmare of finding her daughter’s body in her bedroom.*

This pattern can morph into full-blown symptoms of post-traumatic stress disorder (PTSD) (Stroebe et al., 2001; Kaltman and Bonanno, 2003). It is also important to note that one does not have to have been an actual eye-witness to the suicide to develop trauma symptoms. Instead, one only needs to know the method of the suicide (e.g., gunshot to the head, hanging, etc.) to develop mental imagery about the death scene and dying process. These images then function as trauma “memories.”

These hallmark symptoms of PTSD often require that some type of focused trauma reduction clinical techniques must be used, early in the therapy process, to help the client regain a sense of control over their own reactivity to the horror of the death scene as it was witnessed and/or imagined, and to keep the trauma from “spreading” into other areas of the individual’s physical and psychological life space. I have found that the technique of Eye Movement Desensitization and Reprocessing (EMDR) is one empirically based trauma treatment that can help with trauma reduction in suicide loss survivors, although EMDR is not the only such method for producing relief from trauma symptoms (Kosminsky and McDevitt, 2012; Solomon, 2018). *Other promising and effective treatments for bereavement related PTSD have also been developed* (Cohen et al., 2006; Foa et al., 2009; Pearlman et al., 2014).

• Repair of the Mourner’s Assumptive World

In addition to producing PTSD type symptoms, most traumatic experiences violate the assumptive world of the survivor, particularly their implicit beliefs about safety, predictability, and control in the world (Kauffman, 2002; Currier et al., 2006). One’s assumptive world includes many of the “taken for granted” beliefs that we carry about who we are, who other people are, and what we can expect in terms of our relationships with them. Suicide may violate all of these beliefs for the mourner. For example, I worked with a client whose 15-year old son went upstairs and hung himself after an intense argument with his parents. The parents had no idea that their son was thinking of, let alone capable of, such an act. When I saw the mother 5 years later, she was still struggling with the meaning-making question of “was this really a suicide – or was this an accident?” I asked her what it would mean to her if she could speak with her son again, and he said that he had, in fact, intended to die. My client answered plaintively that “It would mean that I didn’t know my son?” In other words, much of my client’s assumptive world – what she took for granted about her son – was that she knew him well enough to know if he was in danger of suicide; that he would come to her for help if he was wanting to die; and more generally, that she could keep him safe from harm. All of these suppositions were shattered by his sudden suicide. This catastrophe had produced a long-lasting experience of distress, self-doubt, guilt, and anxiety in the client, including a fear that she was a complete failure as a mother, and that she was now in danger of misjudging whether one of her other children might be suicidal. All of this had disrupted her assumptive world, and required therapeutic work on rebuilding those beliefs in a way that would allow her to acknowledge the truth of what had happened, yet permit her to restore a positive self-image and confidence in her functioning as a parent to her other two children.

One of the most important healing tasks for suicide loss survivors is to develop a “bearable” narrative of the suicide, *one that works well enough for the survivor that they can obtain some relief from the “Why?” questions and restore a sense of coherence to their assumptive world. This usually includes construction of a narrative of the death that embraces the complexity of suicide as a kind of “perfect storm” of factors coming together (including the intentions of the deceased) that allowed the suicide to happen, rather than just the simple result of one person’s mistakes or failures.*

This ideally includes a *realistic and fair* explanation of what happened, why it happened, and what responsibility the survivor should *realistically and fairly* assume for the event. Note that this does not mean that all people in a family must agree on all aspects of the story of what happened, or why it happened. Rather, each family member (or person in the social network affected by the death) must develop an explanation of the death that works well enough for them psychologically, and that allows them to begin to reinvest in their life without the deceased (see final task, Reinvestment in Living, below). This task also includes accepting the “blind spots” that are common after suicide, such as the fact that the only person who could answer these “Why?” questions is now dead, and unavailable to offer clarification of their behavior (Sands, 2009; Sands et al., 2011; Neimeyer and Sands, 2017).

• Self-Dosing – Creation of Psychological Sanctuary and Relief from the Pain

The loss of someone to suicide can be excruciatingly painful. Many survivors report that this experience is the deepest psychological anguish they have ever felt. In fact, a recent literature review has confirmed that exposure to the suicide of someone to whom we are psychologically close increases the risk that the person exposed will die by suicide as well (Jordan, 2017). The intense “psychache” (Schneidman, 1981) that suicide leaves in its wake is likely a contributing element to this elevated risk of suicide in survivors. It follows from this that one of the most important integrative tasks is to help the individual cultivate ways to find relief from the despair created by the suicide.

In the beginning, survivors typically do not have any control over their grief – in a very real sense, grief controls the survivor, rather than the other way around. The therapeutic task then becomes one of finding ways to help people more voluntarily regulate this intense pain. This allows the survivor to grieve when they choose to allow this to happen. And correspondingly, when the time and situation is not appropriate, people can usefully learn to “compartmentalize” their grief, so that they can address current challenges in their immediate environment. I have come to label this function as learning to “dose” one’s grief. This idea is captured in the relatively recent model of grieving called the “Dual Process Model of Grief” Dual Process Model (DPM) (Stroebe and Schut, 2010; Stroebe and Schut, 2016). The DPM suggests that in normal, healthy mourning, people oscillate between two orientations toward grief. The first, the Loss Orientation, involves the survivor immersing themselves in the reality of the loss, and the feelings, thoughts, and behaviors that accompany that immersion. The second orientation is termed the Restoration Orientation, and it includes putting the grief on the “back-burner” while the bereaved person learns to cope with the changed world that has been created by the death. Learning to engage in this kind of “flexible attention” to their grief indicates that the mourner is evolving from a primarily involuntary to a more voluntary and “chosen” process of grieving (Kosminsky and Jordan, 2016).

• Development of Social Management Skills

Suicide frequently alters the quantity and quality of social connections between the mourner and their family and larger social networks. For example, in my clinical experience, suicide often strains the relationship between marital partners. People sometimes need to use coping mechanisms that have rarely been employed before in the relationship. To illustrate, after the death of a child to suicide, a mother may seek more or less continuous opportunities to talk about her grief and to process her loss with the child’s co-parent. The partner, in turn, may need to isolate themself and retreat into a defensive “hibernation” stance. This produces a kind of coping asynchrony between the couple, wherein what one person needs to do to integrate the loss is the opposite of what the other needs to do. Moreover, the couple may have little or no experience dealing with this kind of profound emotional dysregulation within and between them. Similarly discrepant reactions can occur between parents and children, between nuclear and extended family members, and even between members of the family and others in their larger social network. Navigating this straining of routine social connections, and managing the usually well-meaning but often clumsy efforts of other people to help by offering platitudes (“they’re in a better place”) or advice (“pray to Jesus”; “take up Yoga”) can add an additional burden of psychological work to be done at a time when the mourner is lacking in skills or energy for such an undertaking. *For example, after the suicide of her husband, a bereaved wife may find spending time with her clergyperson and friends at a Bible study class at her church to be difficult and emotionally draining. Conversations may be guarded for the widow within her church social circle, and discussions of “sin” and “punishment for immoral behavior” within the class awkwardly strained as a result of her husband’s suicide. Indeed, the woman may ultimately feel compelled to leave this church and find another faith community where her background and story are not so much a focus of community members.*

It is important also to note that while in many societies the amount of outright stigma that families encounter after a suicide may be decreasing, suicide and psychiatric disorder nonetheless have a long history of being defamed and punished by most communities. Many suicide loss survivors still experience at least some of the outright avoidance, condemnation, and hostility that have historically been associated with completed suicide (Cvinar, 2005; Botha et al., 2009; Feigelman et al., 2009; Pitman et al., 2018). The social isolation produced by this shunning behavior can negatively affect some people so much that families may attempt to keep the suicide a secret. Or they may experience a rise in family tension and conflict about how to handle these “information management” challenges as a group. In addition, suicide can generate a great deal of anger or rage, some of which may be directed toward the deceased, but much of which may be targeted toward other family members (Jordan and McGann, 2017). These scapegoating processes can seriously erode family cohesiveness, and add to the emotional distress and upheaval with which family members must cope (Jordan et al., 1993). For example, one couple I saw after their young adult son had shot himself presented with enormous anger and hostility between them. The couple had a history of marital conflict, and on many occasions, one of their arguments centered on the father’s refusal to get rid of his gun collection. Finally, the father agreed, but failed to actually dispose of the guns, and the son had subsequently used one of his father’s guns to shoot himself – a fact that infuriated his wife.

• Repair of the Relationship with the Deceased

In a literal sense, death ends the relationship between two people, in least in its previous form. There has been an intellectual “revolution” in modern thanatology, however, with the recognition that for most of human history, most human cultures have allowed, even encouraged, the development of what have been called “continuing bonds” with the deceased (Klass et al., 1996; Klass and Walter, 2001). For example, in traditional Japanese culture, every household would have a shrine or alter to the ancestors in the family, who are symbolically treated as on-going, “living” members of the family system. It is only with the development of 20th century psychiatric thinking that the idea has emerged that mourners are supposed to “decathect” (i.e., withdraw emotional investment in) their bond with the deceased. In addition, the contemporary mental health community has historically judged the failure to do so to be an indicator of unresolved or pathological grief. In contrast, the continuing bonds movement in thanatology has suggested that the task after death is not to “say goodbye” and end the psychological relationship with the deceased. Rather, it could better be characterized as the work of transforming the nature of the attachment to the deceased from one of a relationship in physical reality to one in the psychic/spiritual reality of the mourner (Klass, 1999; Klass and Goss, 1999) – that is, finding a different way to “hold on” to the relationship. The difficulty with the mourning process after a suicide is that suicide often results in a psychological rupturing and betrayal of the relationship between the mourner and the deceased.

The “meaning” of a suicide for a given individual can vary widely from one person to another. Nonetheless, one common theme for suicide loss survivors is to perceive the death as a rejection, abandonment, or even a betrayal by the deceased. Note that how the act of suicide is experienced is “in the eye of the beholder,” and for some survivors, there may be little or no feeling of disloyalty or duplicity by the deceased. But for many people, the death is perceived to be a choice with a critical interpersonal message for the survivor from the deceased about the lack of worth or value of the relationship. All of this can add to the feeling of alienation and estrangement from the deceased.

Continuing bonds theory, as well as common clinical experience, suggests that this kind of sudden breaking of a relationship extracts an even heavier toll from survivors of suicide loss in their mourning process. Correspondingly, an effort by clinicians to help the mourner to reinstate and repair the relationship with the deceased, and to make their peace with the “unfinished business” in the relationship, is often a necessary task of healing after a suicide loss. In my experience, this work often needs to be accomplished by some form of dialogue with the deceased – a dialogue that might have happened if the deceased had betrayed the survivor in some other way, and then was willing to make amends for the damage. The form this dialogue takes can include letter writing to the deceased, empty chair enactment of conversations with the deceased, and other forms of communication between the deceased and the mourner that are experienced as authentic and healing for the bereaved survivor (Jordan, 2012; Neimeyer, 2012, 2016; Botkin, 2014; Neimeyer, in press; Valdez et al., in press).

• Development of a Durable Biography of the Deceased

Walter (1996) has suggested that one of the important tasks of mourning is to create a “durable biography” of the deceased. A durable biography is a narrative of who the deceased was, what they had accomplished, and what they have left behind from their life. He argues that this process is accomplished primarily by a process of shared remembering and storytelling amongst people who knew the deceased. This process typically begins immediately at the time of death (e.g., at the funeral), and may continue on for years, or even generations within family systems. It is a universal and natural way for the community around the deceased to share their grief, and to combine and enrich each mourner’s personal narratives of interaction with the deceased over the course of their life.

Suicide, however, may present special problems for the accomplishment of this important task of mourning. More specifically, because suicide is both a relatively rare and a socially stigmatized cause of death, establishing the communal narrative about the life of the deceased can become either taboo, or alternatively, almost exclusively focused around the mode of death itself. In other words, when someone dies by suicide, that fact may become the only “important” thing about their life story. Thus, mourners who wish to remember the entirety of the life of their friend or relative, not just the manner by which they died, often need to make an extra effort to share (and request that others share) memories that predate and highlight other important aspects of the deceased’s identity and life story. And they also must overcome the social discouragement about talking about and remembering the deceased that comes from others in the interpersonal network, who may view the suicide as a shameful and dishonorable form of death. This implicit prohibition of the “social remembering” aspect of the mourning process can add to the emotional distress and difficulty of the survivors in integrating the loss into their own life narrative as an associate of the deceased.

• Reinvestment in Living

As Sands (2009) and Sands et al. (2011) has pointed out, suicide loss survivors often need to “try on the shoes” of the deceased, but ultimately they must decide to “take off the shoes” of the deceased”. Exposure to suicide, which we know increases the risk of suicide in those who have been exposed (Jordan, 2017), seems to have the effect of raising suicide as an option for the survivor when dealing with life problems and pain, including most importantly the pain of the loss of a loved one to suicide itself. But healing after a suicide loss does require that survivors find reasons to choose to go on with their own life, despite the emotional pain and real life problems that have been left in the wake of the suicide. They also must sometimes contend with a wish for reunion with the deceased that a suicide might produce. For some survivors, their own reasons to go on living may never really be in doubt. But for others, the exposure to the suicide of a loved one creates significant psychological distress, and raises the question of “Why do I want to continue on? What are my reasons for living?” Working through those issues can be a central challenge for many suicide loss survivors, and the therapeutic relationship can be an important crucible for resolving these fundamental existential questions for the survivor.

## Implications for Clinicians

This article has identified a number of ways that grief after suicide may be different in both quality and quantity than grief after other modes of death. I conclude this commentary with some remarks about the implications of these differences for practicing mental health professionals. Again, these recommendations come both from my study of the research literature on suicide bereavement, as well as my own extensive work with suicide loss survivors. These are the “lessons learned” from providing over 40 years of grief therapy for survivors. For additional commentary about the process of providing grief therapy to suicide loss survivors, please see some of these other publications on the topic (Jordan, 2008; Jordan, 2009; Jordan, 2011; Kosminsky and Jordan, 2016).

The first point is that my preferred model for doing the work is longer term therapy. I understand the tremendous pressure within many clinical settings to “speed-up” therapy by providing targeted, but short-term, crisis oriented work for victims of traumatic experiences such as a suicide death. I suspect, however, that this is too often driven by economic, not clinical, concerns. The existing literature suggests that, for people who have been deeply impacted by a suicide, the journey is often long term and transformational (Saarinen et al., 2002; Feigelman et al., 2012; Jordan and McGann, 2017). Therefore, I have come to believe that a “companioning” model of clinical work (Tedeschi and Calhoun, 2003), in which the therapist serves as a transitional attachment figure who helps the bereaved individual re-regulate themselves and integrate the loss over time, is best suited to the nature of the work that needs to be accomplished (Kosminsky and Jordan, 2016). Except at the very beginning of treatment, or in situations where the mourner is in immediate crisis or self-destructive, I believe that it is preferable for the clinician to provide relatively infrequent sessions, but *long term availability* to the mourner.

Second, I believe that, while important in all psychotherapy, the quality of the relationship between therapist and client is of particular importance in helping suicide loss survivors integrate and heal from the loss. Kosminsky and Jordan (2016) have outlined the foundations of an “attachment informed grief therapy” which emphasizes the crucial importance of an on-going, nurturative attachment relationship with a clinician who is empathically attuned to the experience of the client. Rooted in the robust literature on attachment theory and psychotherapy (Wallin, 2007; Berant and Obegi, 2009; Holmes, 2013; Mikulincer et al., 2013; Cozolino and Santos, 2014), this stance is backed by the robust literature on the importance the therapeutic alliance in improving treatment outcomes in all forms of psychotherapy, and of certain therapist behaviors and characteristics that are particularly helpful in fostering that alliance (Norcross and Lambert, 2018). Building on this, Kosminsky and Jordan suggest that certain “Core Capacities,” or necessary interpersonal skills of the grief therapist, are the key to developing a strong therapeutic alliance in work with traumatically bereaved clients. These capacities include the clinician’s skill at creating an emotionally safe relationship, their capacity for empathy, non-defensiveness and repair of alliance ruptures, their ability to tolerate the client’s (and their own) emotional distress without having to “fix-it” immediately, and a deep level of mindfulness and self-knowledge. The reader is referred to the Kosminsky and Jordan (2016) volume for further elaboration and case examples of the application of these Core Capacities to clinical bereavement situations.

Third, I believe that it is important to recognize that grief therapy often involves more than simply helping the client to express and/or gain insight into their feelings. For example, most survivors come to grief therapy with a minimal understanding of psychiatric disorder, suicide, and grief after suicide. Thus, the clinician needs to play an active psychoeducational role in helping the bereaved survivor understand the factors that usually contribute to a suicide and the normality of the intense grief and trauma responses that may follow a suicide. This is important to help the client develop the “bearable narrative” that was mentioned previously.

Likewise, therapy by itself may not be sufficient in providing psychological support for survivors. The opportunity to connect with other suicide loss survivors may be a very helpful experience for many people bereaved by suicide (Feigelman et al., 2008; Hoy, 2016). Opportunities to make their bereavement journey a communal experience can be vital in helping to reduce the sense of shame and isolation that many survivors experience. Sharing with other survivors also allows the bereaved to compare their reactions to those of other survivors, and to learn new adaptive skills by observing the coping efforts of others in similar circumstances. This social exchange can happen in a variety of settings, from face to face peer support groups, to one-on-one interactions with survivors, to new resources that allow survivors to connect with each other online (Beal, 2011; Walker, 2017). Clinicians can be tremendously useful to survivors by helping them find resources beyond the professional mental health community that may provide opportunities for peer support and education about their loss.

Lastly, since a suicide loss can lead to a host of ancillary problems, such as trauma symptoms, existential anxiety about the loss of security and predictability in the world, and disruption of the survivor’s social connections, the therapist must be open to a variety of non-traditional topic areas and clinical techniques that have usually been considered beyond the range of conventional mental health treatment. For example, although many bereaved individuals wonder about the possibility of life after death, this can become an agonizing spiritual question for traditionally religious suicide loss survivors, who may fear for the whereabouts and well-being of their loved one after committing the “sin” of suicide. Some mental health professionals are uncomfortable with discussion of such important issues, having been trained that they are beyond the legitimate concerns of mental health treatment, and, at most, should be referred to a clergy person for “answers.” Likewise, clinicians may also be uncertain about the appropriateness of referral of their clients to psychics or mediums in the community. It is important also for grief therapists to be very mindful of their own beliefs and values about the existential questions that suicide often raises: is there life after death; where do our loved ones go after death; can I communicate with them; and will I be reunited with them? These are necessary and legitimate questions with which survivors struggle and with which therapists should become familiar and comfortable. It is also worth the clinician’s time and effort to learn about developing and unconventional therapy techniques that may help some suicide loss survivors answer such crucial questions (Botkin, 2014; Valdez et al., in press).

## Post-Traumatic Growth After Suicide

Understandably, this article has focused primarily on the negative impact that exposure to a suicide may have on the survivors left behind. As discussed previously, there is now convincing evidence that such exposure can lead to a number of adverse sequelae on survivors, not the least of which is an elevated risk of suicide in the exposed survivor (Jordan, 2017). But it would be a mistake to assume that only bad things can follow the loss of a loved one to suicide. The recognition of what has been called Post-Traumatic Growth (PTG) in survivors after traumatic experiences (Tedeschi and Calhoun, 2008; Calhoun and Tedeschi, 2014) is a new and burgeoning field of inquiry. PTG can be manifested in a changed outlook on life (i.e., an altered assumptive world), greater resilience in the face of stress, and the development of increased prosocial feelings and behavior such as compassion, non-judgmentalness, and hope. Recent research on PTG in suicide loss survivors has suggested that some people show evidence of PTG after suicide (Moore et al., 2015; Genest et al., 2017; Drapeau et al., 2019). And we are beginning to learn some of the correlates of PTG in suicide loss survivors, which will hopefully set the stage for learning what clinicians can do to foster and enhance PTG in people bereaved by suicide. This offers hope for an improving set of methods for mitigating the injurious effects of such a devastating loss, and for promoting the growth of wiser, more compassionate survivors of this painful life experience.

## Author’s Note

Bereavement after a suicide has been neglected in the field of suicidology. This paper reflects the nearly 40 years of clinical work by the author in providing grief therapy to suicide loss survivors. It also draws on his other publications on this topic, as well as the larger literature within thanatology on mourning processes after traumatic death.

## Author Contributions

The author confirms being the sole contributor of this work and has approved it for publication.

## Conflict of Interest

The authors declare that the research was conducted in the absence of any commercial or financial relationships that could be construed as a potential conflict of interest.

## References

[B1] Andriessen K. Krysinska K. Hill N. T. M. Reifels L. Robinson J. Reavley N. (2019). Effectiveness of interventions for people bereaved through suicide: a systematic review of controlled studies of grief, psychosocial and suicide-related outcomes. *BMC Psychiatry* 19:49. 10.1186/s12888-019-2020-z 30700267 PMC6354344

[B2] Beal K. C. (2011). “Parents of suicides-friends & families of suicides internet community,” in *Grief after Suicide: Understanding the Consequences and Caring for the Survivors*, eds Jordan J. R. McIntosh J. L. (New York, NY: Routledge), 381–388.

[B3] Berant E. Obegi J. H. (2009). “Attachment-informed psychotherapy research with adults,” in *Attachment Theory and Research in Clinical Work with Adults*, eds Obegi J. H. Berant E. (New York, NY: Guilford Press), 461–489.

[B4] Bonanno G. A. Neria Y. Mancini A. Coifman K. G. Litz B. Insel B. (2007). Is there more to complicated grief than depression and posttraumatic stress disorder? A test of incremental validity. *J. Abnorm. Psychol.* 116 342–351. 10.1037/0021-843x.116.2.342 17516766

[B5] Botha K.-J. Guilfoyle A. Botha D. (2009). Beyond normal grief: a critical reflection on immediate post-death experiences of survivors of suicide. *AeJAMH* 8 1–11.

[B6] Botkin A. (2014). *Induced After Death Communication: A Miraculous Therapy for Grief and Loss.* Charlottesville, VA: Hampton Roads Publishing.

[B7] Calhoun L. G. Tedeschi R. (2014). *Handbook of Posttraumatic Growth: Research and Practice.* New York, NY: Psychology Press.

[B8] Cohen J. A. Mannarino A. P. Deblinger E. (2006). *Treating Trauma and Traumatic Grief in Children and Adolescents.* New York, NY: Guilford Press, 256.

[B9] Cozolino L. J. Santos E. N. (2014). Why we need therapy - and why it works: a neuroscientific perspective. *Smith Coll. Stud. Soc. Work* 84 155–157.

[B10] Currier J. M. Holland J. M. Neimeyer R. A. (2006). Sense-making, grief, and the experience of violent loss: toward a mediational model. *Death Stud.* 30 403–428. 10.1080/07481180600614351 16610156

[B11] Cvinar J. G. (2005). Do suicide survivors suffer social stigma: a review of the literature. *Perspect. Psychiatr. Care* 41 14–21. 10.1111/j.0031-5990.2005.00004.x 15822848

[B12] De Leo D. Cimitan A. Dyregrov K. Grad O. Andriessen K. (2014). *Bereavement After Traumatic Death: Helping the Survivors.* Boston, MA: Hogrefe.

[B13] Drapeau C. W. Lockman J. D. Moore M. M. Cerel J. (2019). Predictors of posttraumatic growth in adults bereaved by suicide. *Crisis* 40 196–202. 10.1027/0227-5910/a000556 30375239

[B14] Feigelman W. Gorman B. S. Beal K. C. Jordan J. R. (2008). Internet support groups for suicide survivors: a new mode for gaining bereavement assistance. *Omega* 57 217–243. 10.2190/om.57.3.a 18837172

[B15] Feigelman W. Gorman B. S. Jordan J. R. (2009). Stigmatization and suicide bereavement. *Death Stud.* 33 591–608. 10.1080/07481180902979973 19623760

[B16] Feigelman W. Jordan J. McIntosh J. Feigelman B. (2012). *Devastating Losses: How Parents Cope With the Death of a Child to Suicide or Drugs.* New York, NY: Springer.

[B17] Foa E. B. Keane T. M. Friedman M. J. Cohen J. A. (2009). *Effective Treatments for PTSD: Practice Guidelines from the International Society for Traumatic Stress Studies*, 2nd Edn New York, NY: Guilford Press, 658.

[B18] Genest C. Moore M. Nowicke C. M. (2017). “Posttraumatic growth after suicide,” in *Postvention in Action: The International Handbook of Suicide Bereavement Support*, eds Andriessen K. Krysinska K. Grad O. (Cambridge, MA: Hogrefe), 50–59.

[B19] Holmes J. (2013). “Attachment theory in therapeutic practice,” in *Attachment Theory in Adult Mental Health*, eds Danqueth A. N. Berry K. (New York, NY: Routledge), 16–32.

[B20] Hoy W. G. (2016). *Bereavement Groups and Role of Social Support: Integrating Theory, Research, and Practice.* New York, NY: Routledge.

[B21] Jordan J. R. (2001). Is suicide bereavement different? A reassessment of the literature. *Suicide Life Threat. Behav.* 31 91–102. 10.1521/suli.31.1.91.21310 11326773

[B22] Jordan J. R. (2008). Bereavement after suicide. *Psychiatr. Ann.* 38 679–685. 10.3928/00485713-20081001-05

[B23] Jordan J. R. (2009). After suicide: clinical work with survivors. *Grief Matters* 12 4–9.

[B24] Jordan J. R. (2011). “Principles of grief counseling with adult survivors,” in *Grief After Suicide: Understanding the Consequences and Caring for the Survivors*, eds Jordan J. R. McIntosh J. L. (New York, NY: Routledge), 179–223.

[B25] Jordan J. R. (2012). “Guided imaginal conversations with the deceased,” in *Techniques of Grief Therapy: Creative Practices for Counseling the Bereaved*, ed. Neimeyer R. A. (New York, NY: Routledge), 262–265.

[B26] Jordan J. R. (2017). Postvention is prevention—The case for suicide postvention. *Death Stud.* 41 614–621. 10.1080/07481187.2017.1335544 28557579

[B27] Jordan J. R. Kraus D. R. Ware E. S. (1993). Observations on loss and family development. *Fam. Process* 32 425–440. 10.1111/j.1545-5300.1993.00425.x 8163004

[B28] Jordan J. R. McGann V. (2017). Clinical work with suicide loss survivors: implications of the U.S. postvention guidelines. *Death Stud.* 41 659–672. 10.1080/07481187.2017.1335553 28557576

[B29] Jordan J. R. McIntosh J. L. (2011). “Is suicide bereavement different? Perspectives from research and practice,” in *Grief and Bereavement in Contemporary Society: Bridging Research and Practice*, eds Neimeyer R. A. Harris D. L. Winokuer H. R. Thornton G. F. (New York, NY: Routledge), 223–234.

[B30] Jordan J. R. McMenamy J. (2004). Interventions for suicide survivors: a review of the literature. *Suicide Life Threat. Behav.* 34 337–349. 10.1521/suli.34.4.337.53742 15585456

[B31] Kaltman S. Bonanno G. A. (2003). Trauma and bereavement: examining the impact of sudden and violent deaths. *J. Anxiety Disord.* 17 131–147. 12614658 10.1016/s0887-6185(02)00184-6

[B32] Kauffman J. (ed.) (2002). *Loss of the Assumptive World: A Theory of Traumatic Loss. The Series in Trauma and Loss.* New York, NY: Brunner-Routledge, 246.

[B33] Klass D. (1999). *The Spiritual Lives of Bereaved Parents.* Philadelphia, PA: Brunner/Mazel.

[B34] Klass D. Goss R. (1999). Spiritual bonds to the dead in cross-cultural and historical perspective: comparative religion and modern grief. *Death Stud.* 23 547–567. 10.1080/074811899200885 10558614

[B35] Klass D. Silverman P. R. Nickman S. L. (eds) (1996). *Continuing Bonds: New Understandings of Grief. Series in Death Education, Aging, and Health Care.* Philadelphia, PA: Taylor & Francis, 361.

[B36] Klass D. Walter T. (2001). “Processes of grieving: how bonds are continued,” in *Handbook of Bereavement Research: Consequences, Coping, and Care*, eds Stroebe M. S. Hansson R. O. Stroebe W. Schut H. (Washington, DC: American Psychological Association), 431–448. 10.1037/10436-018

[B37] Kosminsky P. S. Jordan J. R. (2016). *Attachment Informed Grief Therapy: The Clinician’s Guide to Foundations and Applications.* New York, NY: Routledge.

[B38] Kosminsky P. S. McDevitt R. (2012). “Eye movement desensitization and reprocessing (EMDR),” in *Techniques of Grief Therapy: Creative Practices for Counseling the Bereaved*, ed. Neimeyer R. A. (New York, NY: Routledge), 95–98.

[B39] Kristensen P. Weisaeth L. Heir T. (2012). Bereavement and mental health after sudden and violent losses: a review. *Psychiatry* 75 75–96. 10.1521/psyc.2012.75.1.76 22397543

[B40] Mauro C. Reynolds C. F. Maercker A. Skritskaya N. Simon N. Zisook S. (2018). Prolonged grief disorder: clinical utility of ICD-11 diagnostic guidelines. *Psychol. Med.* 49 861–867. 10.1017/S0033291718001563 29909789

[B41] McDaid C. Trowman R. Golder S. Hawton K. Sowden A. (2008). Interventions for people bereaved through suicide: systematic review. *Br. J. Psychiatry* 193 438–443. 10.1192/bjp.bp.107.040824 19043143

[B42] Mikulincer M. Shaver P. R. Berant E. (2013). An attachment perspective on therapeutic processes and outcomes. *J. Pers.* 81 606–616. 10.1111/j.1467-6494.2012.00806.x 22812642

[B43] Moore M. Cerel J. Jobes D. A. (2015). Fruits of Trauma? Posttraumatic growth among suicide bereaved parents. *Crisis* 36 241–248. 10.1027/0227-5910/a000318 26440620

[B44] Neimeyer R. A. (in press). *New Techniques of Grief Therapy: Bereavement and Beyond.* New York, NY: Routledge.

[B71] Neimeyer R. A. (ed.) (2012). *Techniques of Grief Therapy: Creative Practices for Counseling the Bereaved. Series in Death, Dying, and Bereavement*. New York: Routledge/Taylor & Francis Group.

[B45] Neimeyer R. A. (ed.) (2016). *Techniques of Grief Therapy: Assessment and Intervention. Series in Death, Dying, and Bereavement.* New York, NY: Routledge.

[B46] Neimeyer R. A. Sands D. (2017). “Suicide loss and the quest for meaning,” in *Postvention in Action: The International Handbook of Suicide Bereavement Support*, eds Andriessen K. Krysinska K. Grad O. (Cambridge, MA: Hogrefe), 71–84.

[B47] Neria Y. Gross R. Litz B. Maguen S. Insel B. Seirmarco G. (2007). Prevalence and psychological correlates of complicated grief among bereaved adults 2.5-3.5 years after September 11th attacks. *J. Trauma. Stress* 20 251–262. 10.1002/jts.20223 17597124

[B48] Norcross J. Lambert M. J. (2018). Psychotherapy relationships that work III. *Psychother. Theory Res. Pract.* 55 303–315. 10.1037/pst0000193 30335448

[B49] Parkes C. M. (2001). Bereavement dissected–A re-examination of the basic components influencing the reaction to loss. *Isr. J. Psychiatry Relat. Sci.* 38 150–156. 11725414

[B50] Parkes C. M. (2013). *Love and Loss: The Roots of Grief and its Complications.* New York, NY: Routledge.

[B51] Pearlman L. A. Wortman C. B. Feuer C. A. Farber C. H. Rando T. A. (2014). *Treating Traumatic Bereavement: A Practitioner’s Guide.* New York, NY: Guilford Press.

[B52] Pitman A. Stevenson F. Osbornab D. J. King M. B. (2018). The stigma associated with bereavement by suicide and other sudden deaths: a qualitative interview study. *Soc. Sci. Med.* 198 121–129. 10.1016/j.socscimed.2017.12.035 29316512 PMC5884304

[B53] Rando T. A. (2015). “When trauma and loss collide: the evolution of intervention for traumatic bereavement,” in *Death, Dying, and Bereavement: Contemporary Perspectives, Institutions, and Practices*, eds Stillion J. M. Attig T. (New York, NY: Springer), 321–334.

[B54] Saarinen P. I. Hintikka J. Lehtonen J. Lönnqvist J. K. Viinamäki H. (2002). Mental health and social isolation among survivors ten years after a suicide in the family: a case-control study. *Arch. Suicide Res.* 6 221–226. 10.1080/13811110214143

[B55] Sands D. C. (2009). A tripartite model of suicide grief: meaning-making and the relationship with the deceased. *Grief Matters* 12 10–17.

[B56] Sands D. C. Jordan J. R. Neimeyer R. A. (2011). “The meanings of suicide: a narrative approach to healing,” in *Grief After Suicide: Understanding the Consequences and Caring for the Survivors*, eds Jordan J. R. McIntosh J. L. (New York, NY: Routledge), 249–282.

[B57] Schneidman E. S. (1981). Postvention: the care of the bereaved. *Suicide Life Threat. Behav.* 11 349–359. 10.1111/j.1943-278x.1981.tb01011.x

[B58] Solomon R. M. (2018). EMDR treatment of grief and mourning. *Clin. Neuropsychiatry* 15 137–150.

[B59] Stroebe M. Schut H. (2016). Overload: a missing link in the dual process model? *Omega* 74 96–109. 10.1177/0030222816666540

[B60] Stroebe M. S. Schut H. (2010). The dual process model of coping with bereavement: a decade on. *Omega* 61 273–289. 10.2190/om.61.4.b 21058610

[B61] Stroebe M. S. Schut H. Finkenauer C. (2001). The traumatization of grief? A conceptual framework for understanding the trauma-bereavement interface. *Isr. J. Psychiatry Relat. Sci.* 38 185–201. 11725417

[B62] Survivors of Suicide Loss Task Force (2015). *Responding to Grief, Trauma, & Distress after a Suicide: U.S. National Guidelines.* Washington, D.C: National Action Alliance for Suicide Prevention.

[B63] Sveen C.-A. Walby F. A. (2008). Suicide survivors’ mental health and grief reactions: a systematic review of controlled studies. *Suicide Life Threat. Behav.* 38 13–29. 10.1521/suli.2008.38.1.13 18355105

[B64] Tedeschi R. G. Calhoun L. G. (2003). *Helping Bereaved Parents: A Clinician’s Guide.* New York, NY: Brunner-Routledge, 188.

[B65] Tedeschi R. G. Calhoun L. G. (2008). Beyond the concept of recovery: growth and the experience of loss. *Death Stud.* 32 27–39. 10.1080/07481180701741251 18652064

[B66] Valdez C. Jordan J. R. Botkin A. (in press). “Induced after death communication,” in *New Techniques of Grief Therapy: Bereavement and Beyond* ed. Neimeyer R. A. (New York, NY: Routledge).

[B67] Walker R. S. (2017). After suicide: coming together in kindness and support. *Death Stud.* 41 635–638. 10.1080/07481187.2017.1335549 28548614

[B68] Wallin D. J. (2007). *Attachment in Psychotherapy.* New York, NY: Guilford Press, 366.

[B69] Walter T. (1996). A new model of grief: bereavement and biography. *Mortality* 1 7–25. 10.1080/713685822

[B70] Wickie S. K. Marwit S. J. (2000). Assumptive world views and the grief reactions of parents of murdered children. *Omega* 42 101–113. 10.2190/2k1c-5qu6-meqn-lx2e

